# FireProt 2.0: web-based platform for the fully automated design of thermostable proteins

**DOI:** 10.1093/bib/bbad425

**Published:** 2023-11-28

**Authors:** Milos Musil, Andrej Jezik, Jana Horackova, Simeon Borko, Petr Kabourek, Jiri Damborsky, David Bednar

**Affiliations:** Loschmidt Laboratories, Department of Experimental Biology and RECETOX, Masaryk University, Brno, Czech Republic; Department of Information Systems, Faculty of Information Technology, Brno University of Technology, Brno, Czech Republic; International Clinical Research Centre, St. Anne’s University Hospital Brno, Brno, Czech Republic; Department of Information Systems, Faculty of Information Technology, Brno University of Technology, Brno, Czech Republic; Loschmidt Laboratories, Department of Experimental Biology and RECETOX, Masaryk University, Brno, Czech Republic; Loschmidt Laboratories, Department of Experimental Biology and RECETOX, Masaryk University, Brno, Czech Republic; Department of Information Systems, Faculty of Information Technology, Brno University of Technology, Brno, Czech Republic; International Clinical Research Centre, St. Anne’s University Hospital Brno, Brno, Czech Republic; Loschmidt Laboratories, Department of Experimental Biology and RECETOX, Masaryk University, Brno, Czech Republic; International Clinical Research Centre, St. Anne’s University Hospital Brno, Brno, Czech Republic; Loschmidt Laboratories, Department of Experimental Biology and RECETOX, Masaryk University, Brno, Czech Republic; International Clinical Research Centre, St. Anne’s University Hospital Brno, Brno, Czech Republic; Loschmidt Laboratories, Department of Experimental Biology and RECETOX, Masaryk University, Brno, Czech Republic; International Clinical Research Centre, St. Anne’s University Hospital Brno, Brno, Czech Republic

**Keywords:** ancestral, back-to-consensus, B-factor, epistasis, evolution, force-field, multiple-point mutant, protein engineering, saturation mutagenesis, thermostability

## Abstract

Thermostable proteins find their use in numerous biomedical and biotechnological applications. However, the computational design of stable proteins often results in single-point mutations with a limited effect on protein stability. However, the construction of stable multiple-point mutants can prove difficult due to the possibility of antagonistic effects between individual mutations. FireProt protocol enables the automated computational design of highly stable multiple-point mutants. FireProt 2.0 builds on top of the previously published FireProt web, retaining the original functionality and expanding it with several new stabilization strategies. FireProt 2.0 integrates the AlphaFold database and the homology modeling for structure prediction, enabling calculations starting from a sequence. Multiple-point designs are constructed using the Bron–Kerbosch algorithm minimizing the antagonistic effect between the individual mutations. Users can newly limit the FireProt calculation to a set of user-defined mutations, run a saturation mutagenesis of the whole protein or select rigidifying mutations based on B-factors. Evolution-based back-to-consensus strategy is complemented by ancestral sequence reconstruction. FireProt 2.0 is significantly faster and a reworked graphical user interface broadens the tool’s availability even to users with older hardware. FireProt 2.0 is freely available at http://loschmidt.chemi.muni.cz/fireprotweb.

## INTRODUCTION

The thermal stability of naturally occurring proteins often limits their applicability in basic research, biotechnology and pharmaceutical industries [1]. Protein engineering has emerged as a promising approach to overcome these limitations by custom tailoring proteins to enhance their usability, even in harsh industrial environments [2, 3]. Random mutagenesis yields reliable results; however, its natural success rate is considerably low for protein stability experiments [4]. In addition, saturation mutagenesis of a significant part of the protein is often not feasible due to the costly and laborious protein characterization [5, 6]. Therefore, there is a growing demand for precise and effective predictors of protein stability.

Several *in silico* tools have been developed for such a task relying on either computationally demanding force-field calculations [7–12], or most notably one or multiple machine-learning techniques [13–19]. Physical effective energy functions are closely related to classical molecular mechanic force-fields allowing for a fundamental analysis of the molecular interactions [20]. Physical force-fields are relatively accurate and capable of predicting the behavior of the proteins under nonstandard conditions such as elevated temperature, nonstandard salinity or nonphysiological pH [21]. However, their versatility is burdened by high computational demands that can be lowered by partially or fully replacing terms in the energy-field equation by statistical potentials derived from the curated datasets of experimental protein structures projected into stability descriptors [22, 23]. While the predictive power of the force-field methods is unmatched by other approaches, their further development is limited by our current level of understanding of the physical and biochemical forces in the protein and the available computational resources [24]. Moreover, the scoring functions neglect or approximate some terms that lower the accuracy of the prediction including protein solvation, dynamics and evaluation of unfolded states necessary for good estimation of the thermodynamic cycle. In addition, force-field methods rely on the availability of high-quality 3D structures that are yet to be well compensated by structure models significantly improved by novel methods like AlphaFold [25].

Machine learning represents a faster alternative to robust force-field calculations that can unearth new features and dependencies unbound by the current knowledge of biochemical forces. The applicability of the machine-learning techniques is strongly dependent on the quality and diversity of training and testing datasets. Several databases [26, 27] and mutation datasets [28] were constructed over the years. However, they contain a relatively low number of mutations with a significant overrepresentation of the destabilizing mutations and an insufficient number of some mutation types, e.g. the charge-changing mutations. Furthermore, these datasets usually do not consider the effect of experimental conditions on protein stability and other characteristics. The pH of the solution has a significant effect on the contribution of the charge–charge interactions that decrease with higher salt concentration [29]. The differences in the experimental techniques can significantly exceed the average experimental error of 0.48 kcal/mol [30]. A large portion of available protein stability data is also measured as a change in melting temperature, while the vast majority of predictive tools estimate the change of Gibbs free energy upon mutation. The free energy of the proteins varies non-linearly with temperature as the stability of proteins generally decreases at lower temperatures [31]. Therefore, thermostability data cannot be well utilized due to the correlation between melting temperature and free energy being about 0.71 [32]. Next to a limited amount of high-quality data, the recent study by Caldararu and coworkers has shown that only a few physico-chemical features significantly affect the predictions of the effect of mutations on protein stability [33].

In recent years, some of the tools have tried to overcome these issues of machine-learning predictors by (i) moving from standard methods such as random forests or support vector machines to convolutional neural networks [34–38] and other deep learning techniques [39], (ii) by utilizing more complex structural features [40, 41] and protein dynamics [42] or (iii) by including temperature-dependent features to be able to predict changes in melting temperature [32]. Meta-predictors also increase the accuracy of the machine-learning predictions by combining multiple tools and models [43, 44], or by completing machine-learning models with statistical potentials [45, 46]. Furthermore, an apparent bias toward the destabilizing mutations caused by the unbalanced dataset [47–49] is being addressed by including reverse mutations in the training datasets [50, 51]. However, most of the tools deal with only single-point mutations, and while several multiple-point predictors [45, 46, 50, 52, 53] have surfaced recently, their limited reliability and potentially antagonistic effect of mutations make it still challenging to utilize these predictions to construct stable multiple-point mutants.

FireProt method [54], initially published in 2015, stands out as one of the few methods [55, 56] that take an opposite approach to the design of stable proteins. Rather than evaluating a set of user-defined mutations, FireProt suggests potentially stabilizing mutations for the target protein. The web version of FireProt [57] made the strategy of automated design of multiple-point mutants accessible to a broader user community, analogously to the PROSS web server, developed by Goldenzweig *et al*. [58]. The new version, FireProt 2.0, retains all the original functionality while introducing several new strategies and user experience improvements. The updated server provides users with two subsets of multiple-point designs. The low-risk designs are subjected to strict filtration of stabilizing mutations based on protein structure, multiple-sequence alignment and physico-chemical properties. The high-risk designs are much more lenient and enforce only position-specific conditions. Both low- and high-risk multiple-point designs are constructed using a novel approach based on the Bron–Kerbosch algorithm [59] minimizing the antagonistic effect among individual mutations.

FireProt 2.0 allows users to limit calculations to a set of user-defined mutations, to run saturation mutagenesis of the entire protein or to select modifications based on B-factors analysis [60]. The fully automated prediction of stabilizing mutations based on ancestral sequence reconstruction [61, 62] is now available as a second evolution-based strategy. FireProt 2.0 also utilizes models of tertiary structures based on deep neural networks downloaded from the AlphaFold database [25] and homology models constructed using ProMod3 [63]. Compared to its predecessor, the updated version is significantly faster, reducing calculation time from over a week to several days. Reworked comprehensive graphical user interface with an integrated Mol* visualizer [64] enhances the tool’s usability for users with older hardware and offers a high degree of variability, enabling users to tailor their calculations to their intended stabilization goals. A vast range of optimization strategies broadens the tool’s usability within the scientific, medical, and industrial communities.

## METHODS

**Figure 1:**
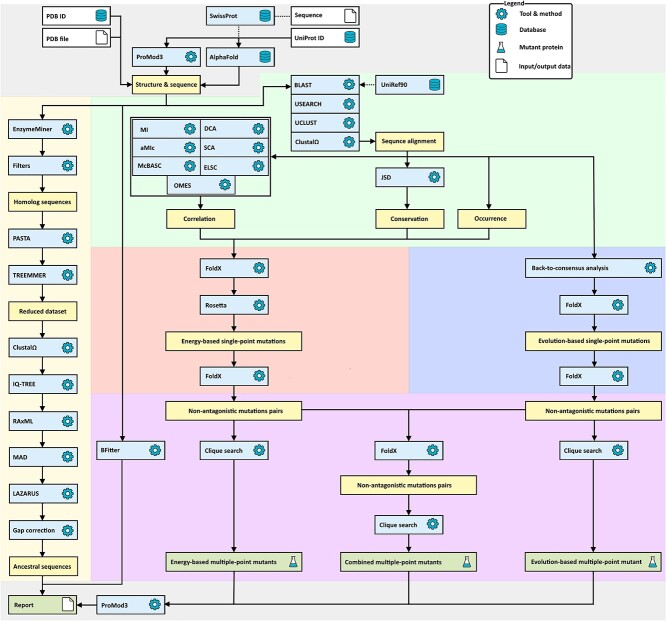
FireProt 2.0 can be started from either protein structure or protein sequence utilized for the construction of the homolog model (gray). Sequence conservation and evolutionary correlation scores are estimated using JSD and the consensus of seven predictive tools, respectively (green). Those annotations serve as filters for the following calculations separated into three main branches identifying potentially stabilizing single-point mutations: (i) ancestral reconstruction employing a full FireProt-ASR workflow (yellow), (ii) force-field predictions using FoldX and Rosetta (red) and (iii) back-to-consensus analysis (blue). In the last step, individual sets of stabilizing single-point mutations are processed in pairs using FoldX, and implementation of the Bron–Kerbosch algorithm is utilized to identify cliques with the best energy evaluations to construct stable multiple-point mutants (purple). Finally, the structures of mutant proteins are modeled by ProMod3 and provided to the user (gray).

FireProt 2.0 uses three main approaches for the identification of potentially stabilizing mutations: (i) energy-based approach, (ii) evolution-based (back-to-consensus), and (iii) ancestral reconstruction-based. The energy-based approach predicts the free-energy change upon mutation by force-field calculations, while the evolution-based approach benefits from well-established back-to-consensus analysis. The ancestral design branch utilizes a fully automated ancestral sequence reconstruction workflow. Prior to the computationally demanding calculations in each approach, several predictive tools and database queries are employed to annotate the protein of interest with various sequence and structural information. This information is then utilized to narrow down the initial set of potential mutations. The entire workflow of the FireProt method is shown in Figure 1.

### Input and annotation of the target protein

The FireProt calculation can be initiated from either the structure or sequence of the target protein. The user can upload a PDB file or provide its PDB ID to provide structural information. The MakeMultimer tool (http://watcut.uwaterloo.ca/tools/makemultimer/) automatically generates the biological assembly, and the user can select the preferred one if multiple biological assemblies are available. In addition, the calculation can be limited to user-defined chains. The user can provide the raw/FASTA amino acid sequence or UniProt [65] ID of the target protein as the sequence input. The AlphaFold database [25] is queried to get a model structure if the UniProt ID is provided. Otherwise, a BLAST [66] search against the PDB database [67] is performed to obtain a structure template for the ProMod3 [63] calculation.

To annotate the protein, the SwissProt database [68] is searched using the protein sequence obtained from the user input or PDB file to remove essential residues from further analysis. Next, the UniRef90 database [69] is searched by the BLAST algorithm to obtain a set of sequence homologs. Sequences with an identity outside of user-defined thresholds (30% and 90% by default) are excluded, and the remaining sequences are clustered using UCLUST [70] with a 90% identity threshold by default. The cluster representatives are sorted based on the BLAST query coverage, and up to 200 sequences are selected to construct a multiple-sequence alignment using Clustal Omega [71]. The conservation coefficient of each residue in the multiple-sequence alignment is estimated by calculating the Jensen–Shannon entropy [72] and amino acid frequencies at the individual positions are stored for filtering in the next steps. Correlated positions are identified using a consensual decision of the OMES [73], MI [74], aMIc [75], DCA [76], SCA [77], ELSC [78] and McBASC [79] methods. Finally, the average B-factor values are computed for all protein residues [80].

### Design of single-point mutations

The FireProt protocol has been expanded to include three branches for identifying potentially stabilizing single-point mutations: (i) energy-based, (ii) evolution-based, and (iii) ancestral reconstruction-based. The protein structure is amended and minimized using FoldX [7] and Rosetta [9] prior to force-field calculations. The filters are applied to exclude potentially deleterious mutations and accelerate the process. Suggested mutations in essential residues, conserved (conservation score 4 or lower) and correlated (consensus score 3.5 or higher by default) positions are considered unsafe and are omitted [81–87]. Additional filters may also be applied, such as disallowing changes to amino acid charges on the protein surface [88] or omitting mutations not present in the multiple-sequence alignment. Saturation mutagenesis is then performed using the FoldX suite (PssmStability module, threshold set for −1 kcal/mol by default), and potentially stabilizing mutations are forwarded to the more comprehensive Rosetta calculations (ddg_monomer module, threshold set for −1.5 kcal/mol by default). The mutations passing both FoldX and Rosetta thresholds are considered for designing multiple-point mutants.

The evolution-based approach identifies potentially stabilizing mutations through the back-to-consensus analysis. Mutations are selected from either the majority or frequency ratio approach. The majority approach discovers mutations at positions where the consensus residue is present in at least 50% of all analyzed sequences. In comparison, the ratio approach lowers the frequency threshold to 40% with the condition that the consensus residue is at least five times more represented than the wild-type amino acid. FoldX evaluates consensus mutations, and the stabilizing ones (threshold set for 0.5 kcal/mol by default) are listed as candidate mutations for designing multiple-point mutants.

In the ancestral reconstruction-based approach, the phylogenetic tree and ancestral sequences are calculated using the FireProt-ASR method [62]. Initially, a reduced set of biologically relevant sequences is aligned, and the phylogenetic tree is constructed. The phylogenetic tree is then rooted using the minimal ancestral deviation algorithm [89], and the ancestral posterior probabilities for individual amino acids are calculated with the Lazarus package [90]. A custom gap correction algorithm is then applied to fill gaps in the reconstructed ancestral sequences [62]. Finally, the ancestral sequence of each internal node between the query sequence and the root of the phylogenetic tree is pair-wise aligned to the query sequence of the target protein, and the substitutions are selected for further analysis as potentially stabilizing mutations.

### Design of multiple-point mutations

FireProt provides users with up to six designs based on the selected analysis. These designs include energy-based, evolution-based and ancestral reconstruction-based approaches and combined mutants constructed from multiple pools of potentially stabilizing mutations. The energy-based and combined mutants are offered in two variants, reflecting the confidence of predictions: (i) high-risk and (ii) low-risk designs. The high-risk mutants are designed for high-risk/high-reward scenarios, allowing for mutations not present in the sequence alignment and charge-changing mutations positioned on the protein surface. However, these mutations can negatively impact protein folding, and their energetic contributions can be difficult to estimate since force-fields can overestimate their effects on stability due to the bias caused by high concentrations of salts in protein crystallization. In contrast, the low-risk designs are constructed with all filters enabled to introduce only the safe mutations. These differentiated scenarios offer flexibility to the users, depending on preferred engineering strategy, allocated resources, access to gene synthesis technologies, availability of infrastructure for experimental validation and experimental conditions. FireProt calculations are processed for the physiological pH of 7, and therefore, high-risk mutations can be easily considered for the experiments conducted under nonphysiological pH or in solutions with high salt concentration [29].

As multiple-point designs cannot be blindly constructed by a combination of individual stabilizing mutations due to the potential antagonistic effects, FireProt minimizes those risks by utilizing FoldX [7] and Bron–Kerbosch algorithm [59]. In the first step, FoldX separately evaluates all pairs of single-point mutations within a 10 Å range for the energy-based and evolution-based approaches using the BuildModel module. Modifications outside of the 10 Å range are considered additive without further analysis. A graph representation of the available mutations is then constructed using the free-energy predictions of individual mutations as nodes and the pair predictions as the edges of the full graph. Antagonistic edges (pairs of mutations with the worse energy compared to the higher stabilizing of the two) are removed from this graph. Bron–Kerbosch algorithm is then used to identify cliques in the graph, and the clique with the highest evaluation of individual mutations is selected for the multiple-point design. Once the energy- and evolution-based designs are completed, the procedure is repeated, considering only the pairs between mutations chosen in previous designs, thus constructing combined multiple-point mutants.

### Description of the web server

#### Input

**Figure 2:**
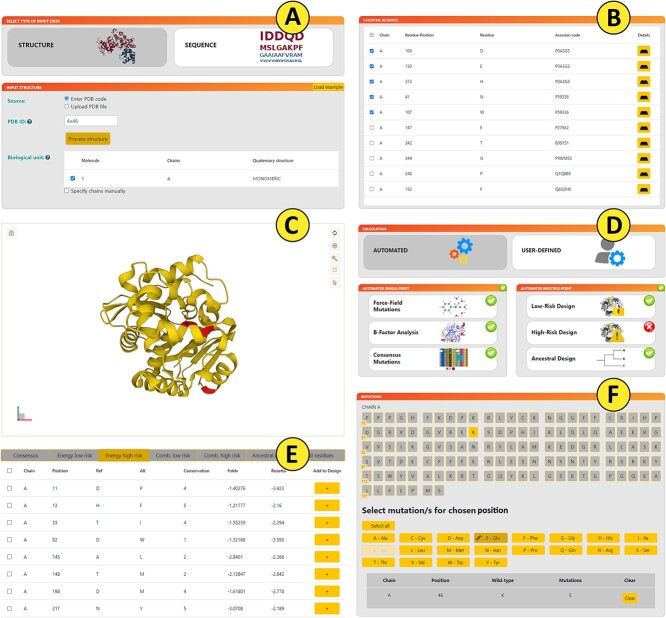
Graphical user interface of FireProt presenting the analysis of the haloalkane dehalogenase DhaA (PDB ID 4E46). (**A**) Input page allowing users to start from either protein sequence or structure. (**B**) The table reporting the essential residues identified in the SwissProt database. (**C**) The Mol* visualization of the wild-type structure of the protein of interest. (**D**) Analysis selection showing options of the fully automated calculation. (**E**) Energy high-risk table providing a list of potentially stabilizing mutations with their conservation scores and free-energy predictions. (**F**) Selection of the positions and mutations in the user-defined mode.

The FireProt web server requires the tertiary structure of the target protein, which can be provided as a PDB ID or a user-defined PDB file (Figure 2A). Alternatively, users can input a custom protein sequence or UniProt identifier to download the structure from the AlphaFold database or build the model by homology modeling. Users can also choose a predefined biological unit generated by MakeMultimer or limit the calculation to the set of manually selected chains.

FireProt offers two mutually exclusive modes—Automated design analyzing the whole protein structure and User-defined analysis of particular positions. The Automated mode (Figure 2D) enables the identification of potentially stabilizing single-point mutations via the Automated single-point panel which enables saturation mutagenesis of all mutable positions, flexibility analysis using B-factors, and back-to-consensus analysis. Users can select low- and high-risk scenarios in the Automated multiple-point panel for the multiple-point designs to be constructed out of the pools of potentially stabilizing single-point mutations. It is also possible to start an ancestral sequence reconstruction analysis from this panel. Alternatively, the User-defined mode (Figure 2F) is intended for users looking to evaluate a specific set of mutations in a shorter period. Multiple mutations can be marked for thorough analysis by all available tools for each selected position.

The user can also select essential residues from the SwissProt database or provide them manually (Figure 2B). The Advanced settings checkbox expands the list of adjustable parameters for (i) construction of the sequence dataset used for multiple-sequence alignment, (ii) identification of consensus residues, (iii) stability thresholds used by FoldX and Rosetta, and (iv) decision threshold employed in the analysis of correlated positions. Advanced mode allows expert users to fine-tune the parameters for their system of interest. However, default values were optimized to provide reliable results in most systems, and it is recommended not to change them in general scenarios.

#### Output

Upon submission, the user is redirected to the output page which can also be accessed later using a unique identifier assigned at the beginning of the calculation. The *Report panel* on the output page informs the user about the status of each step of the FireProt analysis and provides a log of any potential issues encountered during the calculation. Once all calculations are completed, the output page is updated with several new panels, as described below.

### Protein visualization

The web server provides an interactive visualization of the wild-type structure of the target protein using the Mol* library [64] (Figure 2C). This module allows users to switch between different protein visualization styles, analyze protein sequence and highlight selected amino acids in the protein structure. In addition, the Visualization module is connected to the Results panel, which enables users to visualize selected mutations directly in the protein structure.

### Results panel

The results of the FireProt analysis are presented in several tabs based on the set of analyses selected during the input phase (Figure 2E). The All Residues tab provides the available information for each residue. Additional tabs are dedicated to individual multiple-point designs: (i) the Consensus tab presents information from the back-to-consensus analysis, (ii) the Energy low-risk and the Energy high-risk tabs report multiple-point mutants resulting from two respective scenarios of the energy-based approach, (iii) the Combined low- and high-risk tabs presenting a combination of consensus mutations with the low- and high-risk energy-based mutants and (iv) the Ancestral design tab showing single-point mutations obtained from the ancestral sequence reconstruction using the FireProt-ASR method.

### Custom design

The Custom Design panel offers users the possibility to create their multiple-point designs by selecting mutations from the lists available in the individual tabs of the Results panel. After selecting the desired mutations by the plus button, users can generate a custom FASTA sequence by clicking on the Generate sequence button. In addition, users can download all available data related to their FireProt and user-defined designs by clicking on the Download data button. The downloaded .zip archive contains the PDB structure, multiple-sequence alignment, PDF report, raw data in .xml and .csv formats, as well as the FASTA sequences of all FireProt and user-defined designs.

### Validation

To evaluate the robustness of our web server, we have collected an experimental dataset of nine highly mutated proteins from FireProt DB [26] counting 861 mutations (Supplementary Table S1) separated from the original S1573 dataset utilized for FireProt parametrization [57]. Using this dataset, we have calculated sensitivity, specificity and false discovery rates of individual steps of the FireProt workflow and compared them to some of the novel tools for the prediction of the effect of mutations on protein stability (Table 1). While both FoldX and Rosetta show relatively high sensitivity and specificity, their false discovery rates stand at around 50% meaning that about half of the predicted potentially stabilizing mutations are destabilizing. Using the consensual prediction of both FoldX [7] and Rosetta [9], we can observe a notable decrease in sensitivity with every third predicted stabilizing mutation to be a false positive (FP). The FP rate can be completely mitigated by filtering out conserved and correlated residues and by moving the decision threshold from 0 kcal/mol to default values of −1.0 and 1.5 kcal/mol for FoldX and Rosetta, respectively. This comes with the price of significantly lower sensitivity as only about 10% of mutations pass all of the criteria as compared to the FoldX or Rosetta alone. However, low sensitivity with a nullified false discovery rate is the intended goal of the FireProt protocol as the mutations suggested to the user are generally safe to design as multiple-point mutants. In Table 1, we have also compared FoldX and Rosetta with DDGun [50] and DynaMut2 [42] showing still preserving superiority of the force-field approaches over the recent machine-learning tools.

**Table 1:** Evaluation of the individual steps of the energy approach of the FireProt method.

Type	TP	FP	TN	FN	Sensitivity	Specificity	False discovery rate
FoldX	80	94	641	46	0.635	0.872	0.540
Rosetta	68	63	672	58	0.540	0.914	0.481
FoldX + Rosetta	54	27	708	72	0.429	0.963	0.333
+Thresholds	11	0	735	115	0.087	1.000	0.000
+Conservation	7	0	735	119	0.056	1.000	0.000
DDGun	70	153	582	56	0.556	0.792	0.686
DynaMut2	59	86	649	68	0.468	0.883	0.593

The first two lines show the sensitivity, specificity and false discovery rates of FoldX and Rosetta with a decision threshold set at 0 kcal/mol. With FoldX + Rosetta, only the mutations passing both FoldX and Rosetta thresholds are considered stabilizing. The next two lines show the results of the combination of FoldX and Rosetta with decision thresholds set to −1 and 1.5 kcal/mol, respectively, and with including conservation and correlation scores as additional filters. DDGun and DynaMut2 are included for comparison with novel machine-learning tools.

TP, true positives; TN, true negatives; FN, false negatives.

In the next step, we have compared the results of FireProt with PROSS [56], the only directly comparable service for the automated design of multiple-point mutants (Supplementary Table S2). Across nine proteins in the testing dataset, PROSS identified 140 mutations in the total of 81 protein designs (nine designs per protein with different levels of confidence). Out of the 140 mutations, 35 mutations were also identified by FireProt and 22 more positions were suggested with different amino acids. Considering only the top half of the safer designs provided by PROSS, 23 out of 36 mutations were also identified by FireProt. PROSS predictions largely overlap with FireProt’s evolution-based designs as 63 out of 140 mutations (14 out of 36 in the safe designs) are charge-affecting mutations. Charged mutations are generally prohibited in FireProt’s energy-based design as charged residues on the protein surface are often targeted by force-field methods while playing an important role in protein folding [91]. However, charge-affecting mutations are still viable in the FireProt’s high-risk designs and can be commonly found in the consensus design relying on evolution rather than force-field methods. Mutations obtained from the ancestral sequence reconstruction were considered only if the mutation was present in multiple ancestral designs due to the large number of mutations observed close to the root of the phylogenetic tree.

Finally, we have collected over 20 studies from the literature, where the original FireProt method, i.e. equivalent to the Combined high-risk design, was utilized for the protein stabilization (Supplementary Table S3). The optimal temperature of wild-type proteins ranges from 35 to 80°C. Listed studies report an increase of melting temperature by up to 14°C [92] and up to 160-fold improvement of the protein’s half-life [93]. Baseline FireProt protocol was utilized to obtain a highly stabilized haloalkane dehalogenase DhaA by introducing mutations strengthening the interactions around the protein’s access tunnel while preserving its catalytic activity [94]. The ancestral sequence reconstruction algorithm implemented in FireProt 2.0 was also reported to improve the stability of haloalkane dehalogenase by up to 24°C [62]. Furthermore, a recent study by Livada *et al*. [95] evaluated the method on 56 ancestral sequences from which 94% have shown higher or similar stability compared to the wild-type proteins with 56% of the ancestral proteins improving the melting temperature by more than 5°C, with an average improvement of 9°C. Concerning the consensus designs, the proportion of the destabilizing mutations has been estimated to be ~40% among all characterized variants [96, 97].

## CONCLUSIONS

FireProt web [57] is a fully automated tool that enables even inexperienced users without prior knowledge of bioinformatics tools and biological systems of interest to design a set of stable proteins. It eliminates the need to install and set up the individual tools as the default parameters and computational protocols have been optimized to accelerate the calculation while maintaining prediction accuracy. FireProt 2.0 represents a significant advancement in the FireProt method [54], which has already been used to analyze over 6500 proteins since its first release in 2017. This analysis led to a design of more stable mesophilic and thermophilic proteins with increased melting temperature by up to 14°C [92] and up to 160-fold improvement of the protein’s half-life [93]. The new version of the FireProt web was also complemented with the ancestral sequence reconstruction algorithm that was previously utilized for the successful stabilization of haloalkane dehalogenase [62], Renilla luciferase [98] and Ene reductase [95]. Furthermore, users are offered an extended range of available strategies, including low- and high-risk scenarios of the original designs. This feature allows users to fine-tune their risk/reward ratio by enabling filters that make the final designs safer yet more conservative to produce. The previous FireProt version was often used to design and experimentally characterize only single-point mutants. Therefore, FireProt provides all data for the selection of single-point mutations and also information obtained from B-factor analysis [80]. FireProt analysis can now also be started from the protein sequence only, using the AlphaFold database [25] and ProMod3 [63] modeling, which broadens the number of target proteins by several orders of magnitude. The updated graphical user interface, which employs current technologies, has expanded the tool’s availability to users with older hardware, enhancing its usability for the wide range of scientific, medical and industrial communities. Compared to its predecessor, FireProt 2.0 computational demands were dramatically decreased due to the reworked multiple-point mutant construction algorithm from up to a week to less than a day for an average size protein of 300 amino acids. However, the actual calculation time can be longer due to the queue occupancy and the current load on the computational resources. The robustness of the web server was thoroughly tested on a set of 42 diverse proteins.

In the future, FireProt will incorporate new strategies, such as checking the back-bone integrity using ProteinMPNN [99] or removing potentially destabilizing mutations using the novel machine-learning model trained over the manually curated protein stability data from the recently established database FireProt DB [26]. Design of disulfide bridges and reinforcing protein multimerization by the methods like RF Diffusion [100] may further extend the functionalities of the FireProt web server and the charge interactions can be optimized using the methods like TKSA-MC [101–103]. Furthermore, predictions of stabilizing mutations can be combined with designs aiming at improving protein solubility and suppressing protein aggregation, or by considering the changes in the accessible surface area [104]. Those further improvements could potentially mitigate the issue of stability/solubility [88]/activity [105, 106]/heat capacity [107] trade-offs experienced during the designing of practically useful proteins.

**Key Points**

- Protein stability is one of the key determinants of protein applicability. However, most of the computational tools are limited to the prediction of single-point mutants due to the existence of antagonistic effects.
- FireProt 2.0 is a web server offering a one-stop-shop solution for a design of stable multiple-point designs and the prediction of the effect of user-defined mutations.
- FireProt 2.0 builds on top of the original FireProt protocol, retaining its functionality and expanding it with several new features and strategies such as B-factors, conservative low-risk designs and ancestral sequence reconstruction.
- The calculation can now be started from both protein structure or protein sequence using the AlphaFold database and homology modeling implemented in ProMod3.
- Multiple-point designs are now constructed using the novel method based on the Bron–Kerbosch algorithm.

## Supplementary Material

supplementary_table_s1_bbad425

supplementary_table_s2_bbad425

supplementary_table_s3_bbad425

## Data Availability

FireProt 2.0 is a web server available at https://loschmidt.chemi.muni.cz/fireprotweb/. The data used for validation purposes are provided in Supplementary Tables S1–S3 and are available as part of the submission.

## Author Biographies

**Milos Musil** is an assistant professor at the Faculty of Information Technology at Brno University of Technology and a researcher in the field of bioinformatics within Loschmidt's laboratories at the Faculty of Science at Masaryk University. His research is focused on the development of bioinformatics tools and databases, most notably in the area of protein stability and ancestral sequence reconstruction.

**Andrej Jezik** is an undergraduate student of the Faculty of Information Technology at Brno University of Technology. He was involved in the development of FireProt and Caver web.

**Jana Horackova** is a graduate student at the Faculty of Science at Masaryk University. She is interested in the development of bioinformatics tools for the prediction of the effect of mutations on protein stability, and molecular dynamics.

**Simeon Borko** is a software engineer within the bioinformatics team of Loschmidt's laboratories at the Faculty of Science at Masaryk University, where he is involved in the development of several bioinformatics tools including FireProt, PredictONCO, and Caver web.

**Petr Kabourek** is an undergraduate student at the Faculty of Informatics at Masaryk University. He is responsible for the software infrastructure, maintenance of HPC and development of the bioinformatics tools.

**Jiri Damborsky** is the Josef Loschmidt Chair Professor of Chemistry and Professor of Biochemistry at the Faculty of Science at Masaryk University in Brno, Czech Republic and a group leader at the International Centre for Clinical Research. Research of his group focuses on protein engineering, development of new concepts, microfluidic devices and software tools for protein engineering. He is a co-founder of the first biotechnology spin-off from Masaryk University Enantis Ltd.

**David Bednar** is an associate professor and a leader of a group focused on molecular modelling and bioinformatics within Loschmidt's laboratories at the Faculty of Science at Masaryk University. His research interests are in the development of bioinformatics tools and databases, understanding basic principles of enzymology, and designing mutations to improve protein properties.
